# A new method for bedside determination of effective lung volume and functional residual capacity

**DOI:** 10.1113/EP093229

**Published:** 2025-10-13

**Authors:** Andras Gedeon, Jakob Jansson, David Patrickson, Mats Wallin

**Affiliations:** ^1^ Mincor AB Stockholm Sweden; ^2^ Spirotronic AB Stockholm Sweden; ^3^ eHeart AB Stockholm Sweden; ^4^ Department of Physiology and Pharmacology Karolinska Institutet Stockholm Sweden

**Keywords:** carbon dioxide, effective lung volume, functional residual capacity, rebreathing

## Abstract

Established methods of measuring functional residual capacity (FRC) involve sophisticated equipment and elaborate procedures. Here we present a new method based on CO_2_ rebreathing that has a simple fast procedure and only requires end‐tidal CO_2_ monitoring. Ten healthy subjects with diverse anthropometric and respiratory parameters were studied in the sitting position. Reference FRC (RefFRC) and tidal volume (TV) were measured with a Cosmed Quark PFT/DLCO unit using the single‐breath methane dilution technique in combination with spirometry. Rebreathing through an external dead space of precisely known volume and recording the rising end‐tidal CO_2_ value of the first two breaths allows the determination of effective lung volume (ELV) and the calculation of FRC. Two sets of measurements were made on each subject 15 min apart. Bland–Altman analysis of a comparison between FRC and RefFRC showed a mean bias of 0.04 L, with limits of agreement (LoA, 95% CI) of −1.24 to +1.32 L and a percentage error (PE) of 0.54. When the mean value of two observations from a subject (meanFRC) was compared to RefFRC we obtained a mean bias of −0.08 L, LoA (95% CI) of –0.88 to +0.72 L and PE of 0.23. The FRC data obtained demonstrate good absolute accuracy. An average of repeated measurements improves precision indicating that a criterion for exchangeability with the reference method can be met. The simplicity of the equipment and the procedure could make this method attractive in the pre‐operative and the post‐operative settings, as well as in out‐of‐hospital applications.

## Abstract (highlights)

- **What is the central question of this study?**
  Can functional residual capacity (FRC) be determined at bedside using a short CO_2_ rebreathing manoeuvre?
- **What is the main finding and its importance?**
  FRC can be determined in this way, provided that the size of the dead space volume is adequately chosen and is precisely known. The importance of this finding is that such a simple method could allow a more routine use of FRC measurements in pre‐operative assessments and in out of hospital environments, for instance in the management of lung diseases.

## 1 INTRODUCTION

Pre‐operative measurement of functional residual capacity (FRC), particularly in high‐risk patients, is commonly used to help guide anaesthesia but also to predict postoperative pulmonary complications (Ferguson, 1999). Post‐operative monitoring of FRC allows assessment of lung recovery and detection of early pulmonary complications. Although there are several well‐established methods for measuring FRC (Bhakta et al., 2023; Ferguson, 1999; Olsen & Mortensen, 2024; Robinson et al., 2013), they all require expensive, sophisticated equipment and elaborate time‐consuming procedures and none of them can therefore be used routinely at bedside. FRC measurements are also mostly unavailable for primary care management of common pulmonary diseases (Kakavas et al., 2021). Here we describe and evaluate a new method, employing a short period of rebreathing and end‐tidal carbon dioxide monitoring, to measure the effective lung volume (ELV). ELV for carbon dioxide comprises of three compartments, the gas compartment (FRC), the lung tissue compartment and the pulmonary capillary blood compartment. FRC is obtained from ELV using the known relationships between these compartments (Amstrong et al., 1982; Crapo et al., 1982; Hyde et al., 1968).

## 2 METHODS

### 2.1 Ethical approval

This study was approved by the Swedish Ethical Review Authority on 6 May 2024 (registered as No. 2024‐02101‐01, decision based on judgement from a panel of legal and medical professionals). This approval assures conformity with the latest version of the *Declaration of Helsinki* and with other ethical requirements including those on written informed consent and consent to publish as well as data handling and storage procedures.

### 2.2 Physical and respiratory characteristics

Ten subjects, four women and six men, with no known cardio‐pulmonary disease, were recruited and studied in the sitting position. They were between 39 and 70 (mean 55) years old, with weights between 62 and 99 (mean 80) kg; tidal volume (TV) was between 0.58 and 1.7 (mean 1.1) L and respiratory rate (RR) between 4.6 and 16.8 (mean 11) breaths/min. For women average RR and TV was 8.9 breaths/min and 1.2 L and for men 9.7 breaths/min and 1.1 L.

### 2.3 Description of the equipment and the procedure

**FIGURE 1:**
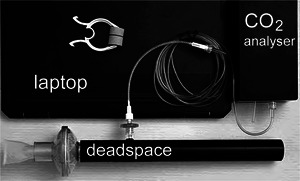
The equipment used for the study.

The equipment used for this study is shown in Figure 1. It was made up of a nose clip, a dead space volume (DV), a sampling infrared (IR) CO_2_ analyser (ET600 Side stream CO2CGM Module Ronseda Electronics Co. Ltd, Shenzhen, China) and a laptop computer for data collection. TV and the functional residual capacity reference (RefFRC) were measured in the sitting position with a Cosmed Quark PFT device with a DLCO module (COSMED SRl, Rome, Italy). The single‐breath methane dilution method (Pesola et al., 2007) was used to measure total lung capacity (TLC), and this combined with spirometry determinations of lung volumes allowed the calculation of RefFRC.

The dead space volume was formed by connecting a mouthpiece, a respiratory filter (ZF‐007, Shandong Zhenfu Medical Device Co. Ltd, Rizhao, China) and a cylindrical pipe. The total volume of the deadspace was determined by an accuracy of better than 3 mL. The sampling port of the analyser was connected to the pipe through a Nafion tube and a sampling filter. DV = 0.148 L was used throughout and resulted in a DV/TV ratio in the range ∼0.10–0.25. The sampling point was ∼10 mm inside the lumen of the pipe.

In order to normalise breathing rates, the subjects were instructed to sit still for about 5 min. Then they were asked to apply the nose clip and to start breathing in the dead space, beginning with an expiration and to breathe in a normal manner till instructed to stop. Data collection took less than 30 s. After sitting still for about 15 min this procedure was repeated for a second set of measurements. FRC and REfFRC were measured by separate persons in separate locations and the results were blinded to each other. RefFRC was measured between the two FRC measurements.

Six subjects could be tested twice, while three measurements could not be reliably evaluated due to irregular breathing and/or leakage at the mouthpiece. Thus, we obtained 17 measurements including six pairs of data points.

In our approach (Gedeon., 2023), first the volume of CO_2_ within the dead space is determined at the end of expiration. The CO_2_ volume in the dead space is calculated as the product of the known dead space volume and a representative sample of the CO_2_ partial pressure within the dead space at the end of expiration.

With DV/TV < 0.25 the CO_2_ partial pressure within the dead pace will decrease slowly and linearly away from the subject. At a sampling point that divides the dead space into two equal parts we can therefore get a representative average value (av*P*_et_) for the entire dead space. The CO_2_ volume is therefore obtained as a product of av*P*_et_ and DV. Introducing this CO_2_ volume into ELV produces an increase in *P*_et_, delta *P*_et_, given by:

$$\mathrm{delta}\;P_{\mathrm{et}}=\frac{\mathrm{av}P_{\mathrm{et}}\times \;\mathrm{DV}}{\mathrm{ELV}}$$

and so

$$\mathrm{ELV}=\frac{\mathrm{av}P_{\mathrm{et}}\times \;\mathrm{DV}}{\mathrm{delta}\;P_{\mathrm{et}}}$$

**FIGURE 2:**
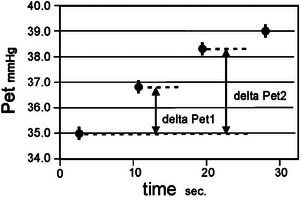
Rebreathing produces a rising *P*_et_ with time.

Rebreathing produces rising *P*_et_ with time as shown in Figure 2, first linearly then gradually approaching a plateau which is reached typically in about 30–40 s, corresponding to a time constant of about 0.06 s^−1^

We consider only the first two breaths, which represent the nearly linear portion of the curve. In principle, the very first breath could determine the value of delta *P*_et_, but in order to get a more robust estimate that is not dependent on one data point only, we use a weighted average of the first two breaths.

The relative weight of the second breath was determined from the estimated time constant so that it took into account that this data point no longer falls entirely on the linear part of the curve.

We calculate delta *P*_et_ from the expression.

$$\mathrm{delta}\;P_{\mathrm{et}}=0.5\times \left(\mathrm{delta}\;P_{\mathrm{et}}1+0.57\times \;\mathrm{delta}\;P_{\mathrm{et}}2\right)$$

and so ELV from:

$$\mathrm{ELV}\;=2.0\times \;\mathrm{av}P_{\mathrm{et}}\times \;\mathrm{DV}/\left(\mathrm{delta}\;P_{\mathrm{et}}1+0.57\times \;\mathrm{delta}\;P_{\mathrm{et}}2\right)$$

In healthy subjects, it has been shown that there is linear relationship between FRC and ELV (Crapo et al., 1982) with the constant of proportionality of 0.82 and so we get:

FRC=0.82×ELV

FRC obtained from this equation is compared to the reference value RefFRC.

### 2.4 Statistics

All statistical calculations were done with the MedCalc Statistical Software (MedCalc Software Ltd, Ostend, Belgium). To show criterion validity for the new method we used the Bland–Altman procedure when comparing to the reference and Student's paired *t*‐test for comparing the two sets of measurements of FRC. An unpaired *t*‐test was used when analysing how women and men compared to reference.

## 3 RESULTS

**FIGURE 3:**
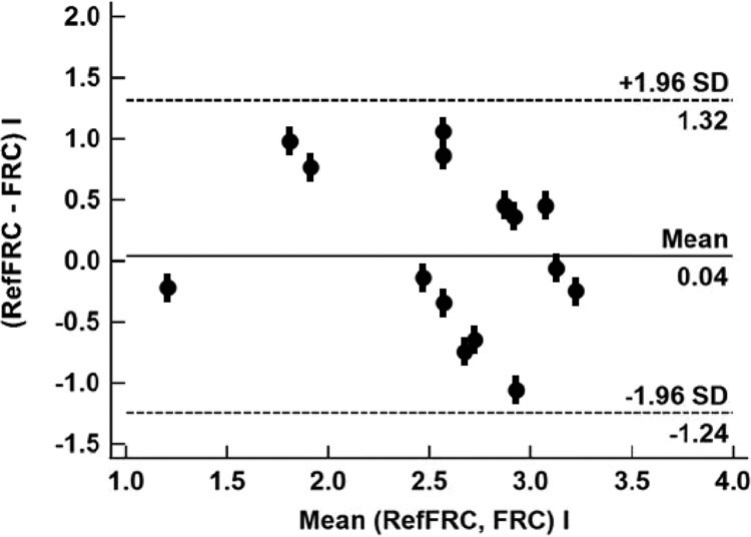
Bland–Altman analysis of FRC versus RefFRC.

Figure 3 shows a Bland–Altman analysis (Bland & Altman, 1986) of a comparison between 17 observations (two identical) of FRC and Ref FRC with mean bias = 0.04 L (LoA, 95% CI: –1.24 to +1.32 L) and PE = 0.54. Comparing the two sets of measurements with a paired *t*‐test we obtained a difference of 0.20 L (LoA, 95% CI: −0.69 to 1.09 L) with *t* = 0.58 and two‐tailed probability *P* = 0.59. This indicates that there is no statistical difference between the two sets.

The differences relative to RefFRC were analysed for women and for men with an unpaired *t*‐test. The result was a difference of −0.17 L (LoA, 95% CI: −1.87 to 1.52 L) with *t* = −0.28 and *P* = 0.79, which shows no statistical sex differences in the accuracy of the method.

**FIGURE 4:**
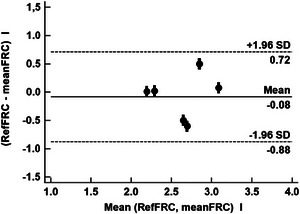
Bland–Altman analysis of meanFRC versus RefFRC.

Figure 4 shows a Bland–Altman analysis of meanFRC versus RefFRC showing a mean bias = −0.08 L (LoA, 95% CI: −0.88 to +0.72 L) and PE = 0.23. Thus, the improved precision allows meanFRC to meet the exchangeability criteria of Critchley (Critchley & Critchley, 1999) with PE < 0.30.

## 4 DISCUSSION

Our method (Gedeon., 2023) is based on first principles (Dalton's law). The only instrument used, the CO_2_ analyser, need not be precisely calibrated because when calculating ELV the gain factor will appear both in the numerator and in the denominator and thus take each other out. Therefore, ELV is determined with about the same absolute accuracy as the dead space volume (DV).

However, FRC depends also on how well the constant of proportionality (*K* = 0.82) in the equation FRC = 0.82 × ELV is known. The value we use is based on a comprehensive study (Crapo et al., 1982) of 90 healthy subjects and is also supported by other work (Amstrong et et al., 1982; Hyde et al., 1968). *K* is determined by the FRC normalised lung tissue volume in which carbon dioxide equilibrates (Crapo et al., 1982; Hyde et al., 1968). Notably, the FRC normalised lung tissue volume is independent of anthropometric parameters as well as age and sex suggesting that it represents a common, intrinsic characteristic across individuals.

To check if *K* = 0.82 is consistent with other expected properties of an average adult lung, consider a typical case with a lung having a surface area of 90 m^2^, a pulmonary capillary blood volume of 100 mL and a tissue volume of 500–700 mL (Hyde et al., 1968). The tissue and blood volume corresponds to a layer on the surface with a mean thickness *h* = 6.7–8.9 µm. If we represent FRC with a single, average sized alveolus, a sphere with radius 125 µm and ELV with the same sphere but covered with a layer with thickness *h* and calculate the ratio between the volumes of the two spheres we get FRC/ELV = 0.79–0.84, which shows that *K* = FRC/ELV = 0.82 is consistent with the characteristics of a typical lung.

Earlier studies that used CO_2_ rebreathing to measure FRC focused on mechanically ventilated patients (Brewer et al., 2011). Contrary to our approach of using the initial rise in CO_2_ (CO_2_ wash‐in), here FRC was determined from the fall of CO_2_ (CO_2_ wash‐out) when returning to normal breathing subsequent to a ∼ 40 s‐long rebreathing period. The FRC values reported had acceptable accuracy and precision compared to both body plethysmography and nitrogen washout reference measurements. These results required stable tidal ventilation and together with the long initial rebreathing period made this approach mainly suitable for patients on controlled ventilation. Nevertheless, they clearly demonstrated the suitability of the rebreathing technique for FRC measurements.

Little is known of whether the equation FRC = 0.82 × ELV can also be used for lungs in disease. However, some indications emerge from a recent study (Sanchez et al., 2024) involving 45 mechanically ventilated patients all with severely reduced FRC where 25 of the patients had COVID‐related ARDS. ELV was measured with capnodynamic methodology (Sanchez et al., 2024) and FRC with computed tomography imaging. One found that the non‐ARDS subjects had *K* ≈ 0.88 while patients with severe ARDS had *K* ≈ 0.77. These results suggest that the value of *K* is only moderately affected by disease, but future studies confirming this are wanted.

We speculate that the modest difference between the two disease groups and that the *K* values for both groups deviated only about 7% from *K* = 0.82 seen in healthy individuals could be understood by remembering that pulmonary capillary blood has a typical volume of 70–90 mL while lung tissue volume is usually around 700 mL, and that the amount of CO_2_ dissolved in lung tissue is about five times that contained in pulmonary capillary blood (Hyde et al., 1968).

The precisely known dead space volume (DV), has a key role in our method. DV should be neither too small nor too large compared to the tidal volume (TV) of the subject tested. If it is too small, then the increase in the *P*_et_ value will be correspondingly too small, leading to reduced precision. If it is too large, then the first two breaths used in the analysis could extend too far beyond the linear portion of the *P*_et_ curve introducing systematic errors and reducing accuracy. Too large a DV can also jeopardise the assumption that the level of carbon dioxide falls off linearly within the dead space and so the sampling point dividing DV into two equal parts may no longer give a representative *P*_et_ value for the whole DV. We have found that if DV is chosen so that the ratio DV/TV is in the range 0.1– 0.25 then these pitfalls are avoided.

The two sets of measurements are shown not to be statistically different. The subjects individually represented a wide range of respiratory parameters, but as a group, women and men were well matched. No statistical difference in the accuracy of the method could be seen for women and men.

The major contributor to the variability in the FRC is irregular breathing. Therefore, careful attention should be given to setting up testing conditions that favour regular breathing. However, precision can be much improved by repeating the measurement and using a mean value instead of a single observation. We find that if two values are averaged, the percentage error is reduced by about a factor of 2, making averaged observations exchangeable with the reference method (Critchley & Critchley, 1999).

In the present study we have chosen to repeat the measurement after 15 min, to ascertain that the gas exchange changes introduced by the first measurement had no influence on the second. However, since only two breaths are needed to obtain the ELV value, the gas exchange balance will be only minimally altered by a measurement and so the rebreathing manoeuvre could have been repeated at least as often as every minute.

### 4.1 Conclusions

FRC is measured with good absolute accuracy when compared to the established reference method of single‐breath methane dilution in combination with spirometry. The measurement takes only a short time (less than 30 s) and can therefore be conveniently repeated. Using the mean value of two observations increases precision so that the present method becomes interchangeable with the reference method.

The simplicity of the procedure and the equipment could make our method attractive both at bedside in the pre‐operative and post‐operative settings and potentially also in out‐of‐hospital environments where a measurement of FRC could add a useful tool when managing lung disease (Kakavas et al., 2021; Moore et al., 2024; Palecek., 2001).

## AUTHOR CONTRIBUTIONS

Andras Gedeon: Conception, design, data analysis and presentation. Jakob Jansson: Technical development and data collection. David Patrickson: Methodology for and measurement of the reference data. Mats Wallin: Expertise in the Fick method, study design and presentation. All authors have read and approved the final version of this manuscript and agree to be accountable for all aspects of the work in ensuring that questions related to the accuracy or integrity of any part of the work are appropriately investigated and resolved. All persons designated as authors qualify for authorship, and all those who qualify for authorship are listed.

## CONFLICT OF INTEREST

A Gedeon is a shareholder and employee of Mincor AB. The other authors have no competing interests, financial or non‐financial, to disclose.

## Data Availability

All data supporting the results are in the paper. All data from this project are stored on two laptops at different locations as approved by the Swedish Ethical Review Authority and can be accessed as Supporting Information by contacting the corresponding author. Ethical and legal requirements as regards identification of the source of the data has been and will be followed.

## References

[eph70070-bib-0001] Amstrong, J. O. , Gluck, E. H. , Crapo, R. O. , Hazel, A. J. , & Hughes, J. M. (1982). Lungtissue volume estimated by simultaneous radiographic and helium dilution methods. Thorax, 37(9), 676–679. 6760448 10.1136/thx.37.9.676 PMC459405

[eph70070-bib-0002] Bhakta, N. R. , McGowan, A. , Ramsey, K. A. , Borg, B. , Kivastik, J. , Knight, S. L. , Sylvester, K. , Burgos, F. , Swenson, E. R. , McCarthy, K. , Cooper, B. G. , García‐Río, F. , Skloot, G. , McCormack, M. , Mottram, C. , Irvin, C. G. , Steenbruggen, I. , Coates, A. L. , & Kaminsky, D. A. (2023). European Respiratory Society/American Thoracic Society technical statement: Standardisation of the measurement of lung volumes, update. European Respiratory Journal, 62(4), 2201519. 37500112 10.1183/13993003.01519-2022

[eph70070-bib-0003] Bland, J. M. , & Altman, D. G. (1986). Statistical methods for assessing agreement between two methods of clinical measurement. Lancet, 327(8476), 307–310. 2868172

[eph70070-bib-0004] Brewer, L. , Orr, J. , Fulcher, E. , & Markewitz, B. (2011). Evaluation of a CO_2_ partial rebreathing functional residual capacity measurement method for use during mechanical ventilation. Journal of Clinical Monitoring and Computing, 25(6), 397–404. 22057246 10.1007/s10877-011-9318-9

[eph70070-bib-0005] Crapo, R. O. , Morris, A. H. , & Gardner, R. M. (1982). Reference values for pulmonary tissue volume, membrane diffusing capacity, and pulmonary. capillary blood volume. Bulletin Européen de Physiopathologie Respiratoire, 18, 893–899. 6927541

[eph70070-bib-0006] Critchley, L. A. H. , & Critchley, J. A. J. H. (1999). A meta‐analysis of studies using bias and precision statistics to compare cardiac output measurement techniques. Journal of Clinical Monitoring and Computing, 15(2), 85–91. 12578081 10.1023/a:1009982611386

[eph70070-bib-0007] Ferguson, M. K. (1999). Preoperative assessment of pulmonary risk. Chest, 115(5), 58S–63S. 10331335 10.1378/chest.115.suppl_2.58s

[eph70070-bib-0008] Gedeon, A. (2023). Swedish Patent SE 545548.

[eph70070-bib-0009] Hyde, R. W. , Puy, R. J. M. , Raub, W. F. , & Forster, R. E. (1968). Rate of disappearance of labeled carbon dioxide from the lungs of humans during breath holding: A method for studying the dynamics of pulmonary CO_2_ exchange. Journal of Clinical Investigation, 47(7), 1535–1552. 5658586 10.1172/JCI105846 PMC297312

[eph70070-bib-0010] Kakavas, S. , Kotsiou, O. S. , Perlikos, F. , Mermiri, F. , Mavrovounis, G. , Gourgoulianis, K. , & Pantazopoulos, I. (2021). Pulmonary function testing in COPD: Looking beyond the curtain of FEV1. Nature Partner Journals Primary Care Respiratory Medicine, 31(1), 23. 10.1038/s41533-021-00236-w PMC8105397 33963190

[eph70070-bib-0011] Moore, A. , Hylton, H. , Long, A. , & Patel, I. (2024). Managing COPD exacerbations in primary care. Drug & Therapeutics Bulletin, 62(7), 102–107. 38950975 10.1136/dtb.2023.000026

[eph70070-bib-0012] Olsen, H. J. B. , & Mortensen, J. (2024). Comparison of lung volumes measured with computed tomography and whole‐body plethysmography—a systematic review. European Clinical Respiratory Journal, 11(1), 2381898. 39081799 10.1080/20018525.2024.2381898 PMC11288198

[eph70070-bib-0013] Palecek, F. (2001). Hyperinflation: Control of functional residual lung capacity. Physiological Research, 50(3), 221–230. 11521732

[eph70070-bib-0014] Pesola, G. R. , Magari, R. T. , Dartey‐Hayford, S. , Coelho‐D'Costa, V. , & Chinchilli, V. M. (2007). Total lung capacity: Single breath methane dilution versus plethysmography in normals. Respirology, 12(2), 291–294. 17298466 10.1111/j.1440-1843.2006.01040.x

[eph70070-bib-0015] Robinson, P. D. , Latzin, P. , Verbanck, S. , Hall, G. L. , Horsley, A. , Gappa, M. , Thamrin, C. , Arets, H. G. , Aurora, P. , Fuchs, S. I. , King, G. G. , Lum, S. , Macleod, K. , Paiva, M. , Pillow, J. J. , Ranganathan, S. , Ratjen, F. , Singer, F. , Sonnappa, S. , … Gustafsson, P. (2013). Consensus statement for inert gas washout measurement using multiple‐ and single‐breath tests. European Respiratory Journal, 41(3), 507–522. 23397305 10.1183/09031936.00069712

[eph70070-bib-0016] Sanchez Giralt, J. A. , Tusman, G. , Wallin, M. , Hallback, M. , Perez Lucendo, A. , Sanchez Galindo, M. , Abad Santamaria, B. , Paz Calzade, E. , Garcia Garcia, P. , Rodriguez Huerta, D. , Canabal Berlanga, A. , & Suarez‐Sipmann, F. (2024). Clinical validation of a capnodynamic method for measuring endexpiratory lung volume in critically ill patients. Critical Care, 28(1), 142. 38689313 10.1186/s13054-024-04928-w PMC11059761

